# Sex Differences in the Characteristics of Acute Aortic Dissection: Stanford Type A Dissection among Elderly Women in Japan

**DOI:** 10.14789/ejmj.JMJ25-0006-OA

**Published:** 2025-10-10

**Authors:** HIROYUKI ISOGAI, SHINSUKE KYOGOKU, TETSURO MIYAZAKI, HIROSHI ABE, MIDORI KAKIHARA, MASAAKI MAKI, RYOSUKE SHIMAI, SHOHEI OUCHI, DAI OZAKI, YUKI YASUDA, FUMINORI ODAGIRI, KAZUHISA TAKAMURA, YOSUKE SAITO, MAKOTO HIKI, KENJI YAGINUMA, MICHIMASA SUZUKI, KATSUHIKO SUMIYOSHI, TAKASHI TOKANO, HIROTAKA INABA, TOHRU MINAMINO, HIROYUKI DAIDA

**Affiliations:** 1 Department of Cardiology, Juntendo Urayasu Hospital, Chiba, Japan; 1 Department of Cardiology, Juntendo Urayasu Hospital, Chiba, Japan; 2 Faculty of Health Science, Juntendo University, Tokyo, Japan; 2 Faculty of Health Science, Juntendo University, Tokyo, Japan; 3 Department of Radiology, Juntendo Urayasu Hospital, Chiba, Japan; 3 Department of Radiology, Juntendo Urayasu Hospital, Chiba, Japan; 4 Laboratory of Bioregulatory Clinical Pharmacology, Faculty of Pharmacy, Juntendo University, Chiba, Japan; 4 Laboratory of Bioregulatory Clinical Pharmacology, Faculty of Pharmacy, Juntendo University, Chiba, Japan; 5 Department of Cardiovascular Surgery, Juntendo Urayasu Hospital, Chiba, Japan; 5 Department of Cardiovascular Surgery, Juntendo Urayasu Hospital, Chiba, Japan; 6 Department of Cardiovascular Biology and Medicine, Juntendo University School of Medicine, Tokyo, Japan; 6 Department of Cardiovascular Biology and Medicine, Juntendo University School of Medicine, Tokyo, Japan; 7 Department of Health and Nutrition, Faculty of Human Science, Tokiwa University, Ibaraki, Japan; 7 Department of Health and Nutrition, Faculty of Human Science, Tokiwa University, Ibaraki, Japan

**Keywords:** aortic dissection, gender, aging, malnutrition, renal dysfunction

## Abstract

**Objectives:**

Acute aortic dissection (AAD) still remains a life-threatening medical emergency. However, only a few data exist on sex-related differences in patients with AAD over the recent years.

**Materials and Methods:**

The medical records of 192 consecutive patients admitted to Juntendo University Urayasu Hospital from January 2008 to December 2021 who had been diagnosed with AAD were retrospectively collected. Thereafter, sex-related differences in patient characteristics, type of dissection, maximal diameters of dissection, the onset-to-arrival time, and short-term mortality were determined.

**Results:**

A total of 164 patients were ultimately enrolled in this study. Compared to men, women were significantly older, were less obese, and had lower prevalence of metabolic syndrome. A significantly higher proportion of women than men underwent Stanford type A dissections (52% vs. 25%), except among those aged 50 years and younger. Women had significantly larger maximal aortic dissection diameters compared to men (43.3 ± 9.5 vs. 38.9 ± 7.2 mm). Women with type A dissection were older, had lower body mass index and albumin levels compared to men, and demonstrated poorer renal function than women with type B dissection.

**Conclusion:**

In the context of Japan's aging society, older, underweight women with chronic kidney disease and malnutrition may represent a high-risk population for Stanford type A aortic dissection.

## Introduction

Studies have shown that acute aortic dissection (AAD) still remains a life-threatening medical emergency associated with high mortality rates^1-6)^. The International Registry of Acute Aortic Dissection (IRAD), which included patients with acute aortic dissections from 1996 to 2001, had reported sex-related differences in clinical presentation, diagnosis, management, and outcomes^7)^. Owing to the recent development of an effective and safe transfer system, advancements in diagnostic imaging and surgical techniques, and rapid aging of society, dramatic changes in the clinical features and prognosis of AAD can be expected. However, limited data have been available on sex-related differences in patients with AAD occurring over the recent years in developed countries. The current study therefore aimed to investigate sex-related differences in patients with AAD diagnosed within the last 10 years in an urban Japanese area.

## Material and Methods

### Study population

Medical records of 192 consecutive patients admitted to Juntendo University Urayasu Hospital from January 2008 to December 2021 who had been diagnosed with AAD according to European Society of Cardiology guideline of aortic disease were then retrospectively collected^8)^. After excluding 28 patients diagnosed with other diseases (i.e., 9 with chronic aortic disease, 5 with rupture of aortic aneurysms, 3 with ischemic heart disease, 3 with pulmonary thromboembolisms, 2 with aortic injury due to traffic accidents, 2 with pneumonia, 1 with pancreatitis, and 4 with undiagnosed back pain), 164 patients were ultimately enrolled herein. Informed consent was obtained through an opt-out process on the website. Those who declined were excluded. This study was approved by the Institutional Review Board of Juntendo University Urayasu Hospital (assignment number: U21-0017).

### Data collection

Evaluated patient data included age, sex, body mass index (BMI), blood pressure (BP) on admission, present illness, past history, history of smoking, laboratory data on admission, results of urgent contrast-enhanced computed tomography (CT), onset-to-arrival time, in-hospital death, and 30-day mortality. Hypertension was defined based on prior diagnosis of hypertension (systolic BP > 140 mmHg or diastolic BP > 90 mmHg) or current treatment with antihypertensive agents. Diabetes mellitus was defined based on prior diagnosis of diabetes mellitus, hemoglobin A1c (HbA1c; national glycohemoglobin standardization program calculation) levels ≥ 6.5%, or current treatment with antidiabetic agents or insulin. Dyslipidemia was defined based on prior diagnosis of dyslipidemia, presence of abnormal lipid profile [i.e., triglyceride (TG) levels ≥ 150 mg/dL, low-density lipoprotein cholesterol (LDL-C) ≥ 140 mg/dL, or high-density lipoprotein cholesterol (HDL-C) < 40 mg/dL], or current treatment with antidyslipidemic agents. Subjects with metabolic syndrome were defined as having a BMI of ≥ 25 plus two or more of the following factors: (1) elevated TG (≥ 150 mg/dL) and/or reduced HDL-C (< 40 mg/dL) concentrations or specific treatment for such lipid abnormalities; (2) hypertension; and (3) diabetes mellitus.

### Definition of acute aortic dissection and measurement of aortic dissection diameter

All 164 patients had undergone urgent contrast- enhanced CT on admission. All CT angiographies were performed on multidetector row CT scanners [Aquilion 16 (Toshiba, Tokyo, Japan) from 2008 to 2018; Aquillion 64 (Toshiba, Tokyo, Japan) from 2008 to 2018; Siemens Somatom Definision Flash (Siemens Healthineers, Erlangen, Germany) from 2013 to 2020; Aquillion One Genesis (Cannon, Tokyo, Japan) from 2018 to 2020; or Aquilion Lightning Herios (Cannon, Tokyo, Japan) from 2018 to 2020]. All CT images were reviewed on workstations [Zio Station (Ziosoft, Inc, Tokyo, Japan) from 2008 to 2010 and Synapse Vincent (Fujifilm, Tokyo, Japan) from 2011 to 2020]. The presence of an aortic dissection was defined as confirmed true and false lumens more than 1 cm in length on contrast- enhanced CT. The maximal aortic dissection diameter was re-evaluated by an independent researcher, with the obtained measurements perpendicular to the centerline of aorta.

### Statistical analysis

Continuous variables are presented as mean ± standard deviation or as medians with interquartile ranges (25th-75th percentiles), while categorical variables are expressed as percentages. Significant differences between groups were analyzed using unpaired Student’s *t*-test, the Mann-Whitney-Wilcoxon rank-sum test, the chi-square test, or Fisher’s exact test as appropriate. Simple linear regression analysis was conducted to assess the relationship between age and the maximum diameter of the dissection. Patients were then divided into two groups according to type of Stanford dissection, after which interactions between sex differences and type of Stanford dissection were calculated. All statistical analyses were conducted using JMP (version 12.0 for Macintosh, SAS Institute, Cary, NC, USA), with P values < 0.05 indicating statistical significance.

## Results

### Differences in baseline characteristics according to sex

The baseline characteristics of the included patients are summarized in Table 1. Compared to men, women were significantly older, were less obese, and showed significantly lower systolic and diastolic BPs. Women also had lower prevalence of metabolic syndrome and current smokers. No significant differences in the prevalence of hypertension, diabetes mellitus, dyslipidemia, family history of cardiovascular disease, and Marfan syndrome was observed according to sex. Women had significantly lower white blood cell counts, hemoglobin, total protein, albumin, serum creatinine, γ-glutamic pyruvic transaminase (GTP) and TG compared to men. No significant differences in onset-to-arrival time and mortality were observed according to sex.

**Table 1:** Characteristics of subjects with acute aortic dissection

	All (n = 164)	Women (n = 54)	Men (n = 110)	P
Age (years)	67 ± 13	**71 ± 12**	**66 ± 13**	**0.013**
Body mass index (kg/m^2^)	24.0 ± 4.5	**21.6 ± 4.1**	**25.1 ± 4.3**	**<0.001**
Systolic BP (mmHg)	148 ± 40	**134 ± 36**	**154 ± 41**	**0.002**
Diastolic BP (mmHg)	83 ± 24	**76 ± 21**	**86 ± 25**	**0.023**
Heart Rate (bpm)	78 ± 17	78 ± 15	77 ± 18	0.746
Hypertension (n, %)	123 (75)	39 (72)	84 (76)	0.567
Diabetes mellitus (n, %)	17 (10)	4 (7)	13 (12)	0.586
Dyslipidemia (n, %)	46 (28)	20 (37)	26 (24)	0.076
Current smoker (n, %)	60 (37)	**7 (13)**	**53 (48)**	**<0.001**
Family history of CVD (n, %)	29 (18)	10 (19)	19 (17)	0.804
Marfan syndrome (n, %)	2 (1)	1 (2)	1 (1)	0.552
Metabolic syndrome (n, %)	26 (16)	**2 (4)**	**24 (22)**	**0.003**
Number of risks of Mets	1.7 ± 1.0	**1.3 ± 0.8**	**1.8 ± 1.0**	**0.002**
**Laboratory data**				
White blood cell (10^2^/μL)	90 ± 33	**82 ± 35**	**94 ± 32**	**0.005**
Hemoglobin (g/dL)	13.3 ± 2.1	**11.7 ± 1.5**	**14.0 ± 1.9**	**<0.001**
Platelets (10^4^/μL)	21.1 ± 7.8	21.8 ± 10.0	20.8 ± 6.6	0.939
Total protein (g/dL)	6.7 ± 0.7	**6.5 ± 0.7**	**6.8 ± 0.7**	**0.010**
Albumin (g/dL)	3.6 ± 0.5	**3.5 ± 0.5**	**3.7 ± 0.5**	**<0.001**
Creatinine (mg/dL)	0.8 (0.7, 1.1)	**0.7 (0.6, 0.9)**	**0.9 (0.8, 1.2)**	**<0.001**
eGFR (mL/min)	62.8 ± 25.5	64.2 ± 25.2	62.1 ± 25.7	0.588
AST (IU/L)	32 ± 34	39 ± 40	29 ± 30	0.356
ALT (IU/L)	27 ± 29	27 ± 34	27 ± 27	0.057
γ-GPT (IU/L)	50 ± 72	**45 ± 89**	**53 ± 62**	**0.002**
Total cholesterol (mg/dL)	177 ± 38	180 ± 37	176 ± 39	0.446
HDL cholesterol (mg/dL)	50 ± 18	55 ± 22	48 ± 15	0.068
LDL cholesterol (mg/dL)	109 ± 31	106 ± 32	110 ± 30	0.496
Triglycerides (mg/dL)	117 ± 77	**98 ± 48**	**127 ± 86**	**0.027**
Hemoglobin A1c (NGSP, %)	5.9 ± 0.6	5.7 ± 0.4	5.9 ± 0.6	0.186
Glucose on admission (mg/dL)	142 ± 56	140 ± 55	143 ± 56	0.555
C-reactive protein (mg/dL)	2.5 ± 5.0	3.4 ± 6.4	2.1 ± 4.1	0.577
d-dimer (μg/mL)	15.4 ± 33.9	9.3 ± 11.1	18.5 ± 40.6	0.168
**Onset-to-arrival time (n, %)**				
<1 hour	57 (35)	17 (31)	40 (36)	0.536
<4 hours	109 (66)	34 (63)	75 (68)	0.508
<12 hours	118 (72)	38 (70)	80 (73)	0.753
<24 hours	128 (78)	44 (81)	84 (76)	0.452
**Mortality**				
In-hospital death (n, %)	8 (5)	1 (2)	7 (6)	0.274
30-day mortality (n, %)	6 (4)	1 (2)	5 (5)	0.665

Data are presented as means ± SD or as medians with interquartile ranges (25th-75th percentiles). BP = blood pressure, CVD = cardiovascular disease, eGFR = estimated glomerular filtration rate, AST = aspartate aminotransferase, ALT = alanine aminotransferase, GPT = glutamic pyruvic transaminase, HDL = high-density lipoprotein, LDL = low-density lipoprotein. Bold means P < 0.05 among sexes.

### Differences in features of aortic dissection according to sex

**Figure 1:**
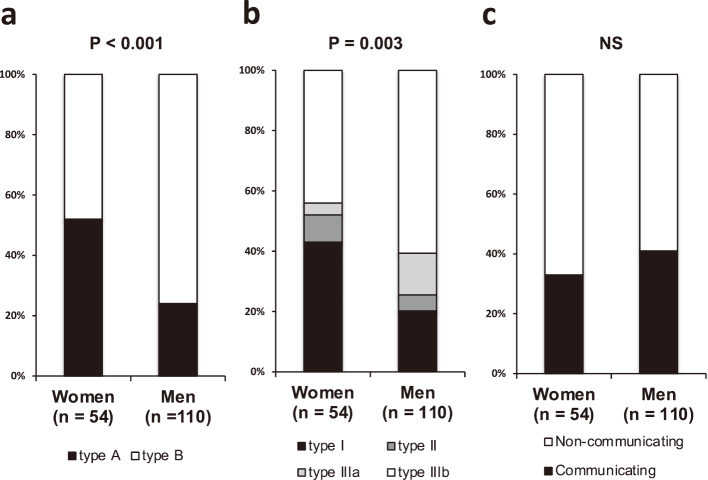
Women had a significantly higher prevalence of Stanford type A dissections compared to men (a). Women had a higher prevalence of DeBakey type I dissection, whereas men had a higher prevalence of DeBakey type IIIb dissection (b). No significant sex differences in the prevalence of communicating aortic dissection were observed (c).

**Figure 2:**
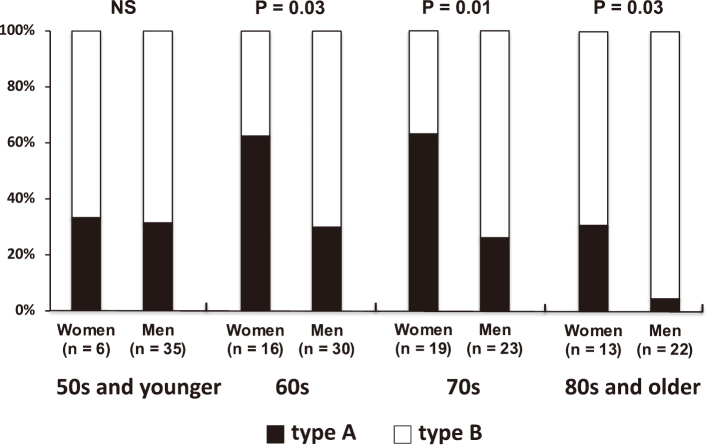
Women had a significantly higher proportion of Stanford type A dissection camped to men, except for subjects aged 50 years and younger.

**Figure 3:**
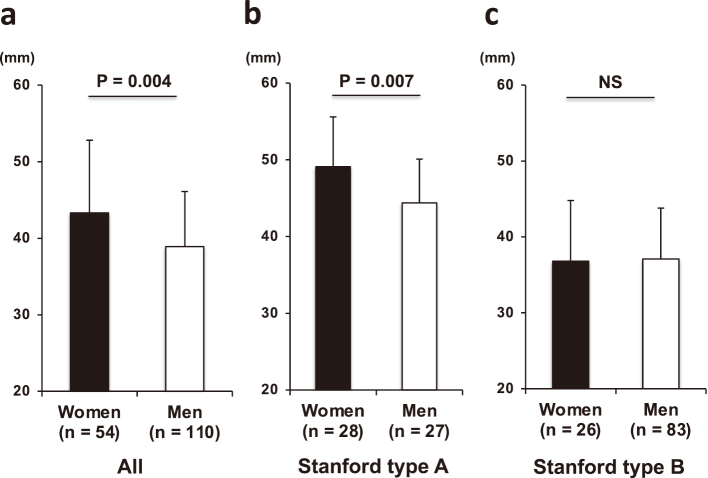
Women had significantly larger maximal aortic dissection diameters compared to men (a). Among patients with Stanford type A dissection, women still had significantly larger maximal diameters compared to men (b), whereas no such differences were observed in patients with Stanford type B dissection (c).

Women showed a significant higher prevalence of Stanford type A dissections (52% vs. 25%, P < 0.001; Figure 1a) and DeBakey type I dissections (43% vs. 19%, P < 0.001; Figure 1b) compared to men. No significant difference in the prevalence of communicating aortic dissections was noted between women and men (33% vs. 41%; Figure 1c). Women had a significantly higher proportion of Stanford type A dissections compared to men, except among subjects aged 50 years and younger. Among those aged 60 and 70 years, women had a higher proportion of type A dissections (60%) compared to men (approximately 30%). Although the proportion of Stanford type A dissections in women aged 80 years and older decrease to approximately 30%, this figure still remained significantly higher than that for men (Figure 2). Women had significantly larger maximal aortic dissection diameters compared to men (43.3 ± 9.5 vs. 38.9 ± 7.2 mm, P = 0.004; Figure 3a). Additionally, in women with type B dissection, the maximum diameter of the dissection tended to increase with age (r = 0.433, P = 0.035). In contrast, no significant age-related changes were observed in women with type A dissection or in men with either type A or type B dissection. Among patients with Stanford type A dissection, women still had significantly larger maximal aortic dissection diameters compared to men, whereas no significant sex differences in maximal diameter were observed among those with Stanford type B dissection (49.1 ± 6.5 vs. 44.4 ± 5.7 mm, P = 0.007, 36.8 ± 8.0 vs. 37.1 ± 6.7 mm, P = 0.882, P for interaction = 0.043; Figure 3b, 3c).

### Sex differences in patients with Stanford type A and B dissections

Women with Stanford type A dissection were significantly older, were less obese, and had lower prevalence of metabolic syndrome and current smokers compared to their male counterparts (Table 2). Women with type A dissection also showed significantly lower white blood cell counts, hemoglobin, serum creatinine, TG and d-dimer and higher levels of total cholesterol and HDL-C compared to their male counterparts. Similar to those with type A dissection, women with Stanford type B dissection were less obese and had a lower prevalence of current smokers compared to their male counterparts. Women with Stanford type B dissection showed significant lower levels of systolic blood pressure, hemoglobin, albumin, serum creatinine, alanine aminotransferase (ALT), and γ- glutamic pyruvic transaminase (GTP) compared to their male counterparts. No significant sex differences in the onset-to-arrival time and mortality were in both type A and B dissection.

Regardless of sex, patients with Stanford type A dissection showed significantly lower systolic and diastolic blood pressures. Women with Stanford type A dissection had significantly lower levels of platelets, estimated glomerular filtration rate (eGFR), and C-reactive protein (CRP) and higher levels of serum creatinine and glucose compared to women with Stanford type B dissection, whereas men with Stanford A dissection showed significantly lower levels of total protein, albumin, total cholesterol, and HDL-C and higher levels of aspartate aminotransferase (AST), ALT, glucose and d-dimer compared to men with Stanford type B dissection. Men with Stanford type A dissection showed significantly higher proportions of onset-to-arrival times less than 1, 4, and 12 hours compared to men with type B dissection. Women with type A dissection showed significantly higher proportions of onset-to-arrival time less than 12 hours compared to women with type B dissection, but not for shorter time intervals. There were no significant differences in the onset-to-arrival times among the age groups (≤ 50s, 60s, 70s, and ≥ 80s; P = 0.358). Additionally, regardless of gender, patients with an onset-to-arrival time of 24 hours or more had significantly higher CRP levels compared to those who arrived within 24 hours (Women: 1.8 ± 4.8 vs. 10.3 ± 8.1 mg/dL, P = 0.004; Men: 0.8 ± 1.9 vs. 6.2 ± 6.1 mg/dL, P < 0.001). Stanford type A dissection had significantly higher mortality rates than type B dissection in only men. Statistical analysis for interactions between sex and type of dissection revealed that women with Stanford type A dissection had higher white blood cell counts and TG compared to their male counterparts.

**Table 2:** Sex differences in study subjects characteristics according to type of dissection

	Stanford type A		P	Stanford type B		P	P for interaction
	Women (n = 28)	Men (n = 27)		Women (n = 26)	Men (n = 83)		
Age (years)	**71 ± 10**	**62 ± 11**	**0.002**	71 ± 14	67 ± 13	0.147	0.384
Body mass index (kg/m^2^)	**21.4 ± 3.9**	**26.3 ± 4.2**	**<0.001**	**21.8 ± 4.3**	**24.8 ± 4.2**	**0.003**	0.184
Systolic BP (mmHg)	**119 ± 36**	**121 ± 36**	0.866	151 ± 28**	165 ± 36**	**0.043**	0.322
Diastolic BP (mmHg)	**69 ± 21**	**68 ± 17**	0.978	85 ± 16**	91 ± 25**	0.218	0.311
Heart rate (bpm)	79 ± 16	74 ± 20	0.215	77 ± 14	78 ± 18	0.699	0.264
Hypertension (n, %)	20 (71)	17 (63)	0.503	19 (73)	67 (81)	0.414	0.295
Diabetes mellitus (n, %)	2 (7)	2 (7)	1.000	2 (8)	11 (13)	0.730	0.664
Dyslipidemia (n, %)	8 (29)	4 (15)	0.329	12 (46)	22 (27)	0.065	0.968
Current smoker (n, %)	**4 (14)**	**13 (48)**	**0.008**	**3 (12)**	**40 (48)**	**0.001**	0.853
Family history of CVD (n, %)	6 (21)	3 (11)	0.467	4 (15)	16 (19)	0.778	0.251
Marfan syndrome (n, %)	0 (0)	0 (0)	-	1 (4)	1 (1)	0.422	1.000
Metabolic syndrome (n, %)	**1 (4)**	**8 (30)**	**0.024**	1 (4)	16 (19)	0.067	0.700
Number of risks of Mets	**1.1 ± 0.9**	**2.1 ± 0.9**	**0.002**	1.4 ± 0.8	1.8 ± 0.9	0.178	0.083
**Laboratory data**							
White blood cell (10^2^/μL)	**73 ± 20**	**99 ± 37**	**0.002**	92 ± 44	93 ± 31	0.397	**0.026**
Hemoglobin (g/dL)	**11.7 ± 1.5**	**13.7 ± 2.0**	**<0.001**	**11.6 ± 1.5**	**14.1 ± 1.8**	**<0.001**	0.405
Platelets (10^4^/μL)	**19.2 ± 7.5**	19.3 ± 6.9	0.940	24.6 ± 11.5*	21.2 ± 6.4	0.311	0.206
Total protein (g/dL)	6.4 ± 0.7	**6.3 ± 1.0**	0.989	6.7 ± 0.6	7.0 ± 0.5**	0.052	0.134
Albumin (g/dL)	3.4 ± 0.4	3.5 ± 0.5	0.148	**3.5 ± 0.6**	3.8 ± 0.4**	0.008	0.187
Creatinine (mg/dL)	**0.8 (0.6, 0.9)**	**1.0 (0.8, 1.3)**	**0.002**	0.6 (0.5, 0.7)*	**0.9 (0.8, 1.1)**	**<0.001**	0.088
eGFR (mL/min)	**57.5 ± 26.5**	56.2 ± 19.2	0.699	71.5 ± 21.8*	64.1 ± 27.3	0.167	0.490
AST (IU/L)	51 ± 51	**44 ± 55**	0.920	26 ± 16	24 ± 13*	0.798	0.658
ALT (IU/L)	36 ± 45	**41 ± 42**	0.165	**17 ± 11**	23 ± 17*	**0.046**	0.857
γ-GPT (IU/L)	41 ± 50	50 ± 43	0.130	**49 ± 120**	**54 ± 67**	**0.013**	0.880
Total cholesterol (mg/dL)	**178 ± 40**	**155 ± 40**	**0.044**	183 ± 36	183 ± 36**	0.970	0.094
HDL cholesterol (mg/dL)	**57 ± 23**	**43 ± 10**	**0.011**	52 ± 21	50 ± 16*	0.796	0.079
LDL cholesterol (mg/dL)	104 ± 31	102 ± 31	0.782	108 ± 33	112 ± 30	0.547	0.539
Triglycerides (mg/dL)	**90 ± 39**	**167 ± 136**	**0.027**	105 ± 55	115 ± 58	0.367	**0.015**
Hemoglobin A1c (NGSP, %)	5.6 ± 0.4	5.7 ± 0.5	0.626	5.8 ± 0.4	6.0 ± 0.7	0.687	0.593
Glucose on admission (mg/dL)	**161 ± 66**	**167 ± 73**	0.705	118 ± 29**	135 ± 48*	0.056	0.561
C-reactive protein (mg/dL)	**1.6 ± 3.5**	1.3 ± 3.5	0.940	5.3 ± 8.2*	2.3 ± 4.3	0.095	0.123
d-dimer (μg/mL)	**11.8 ± 12.1**	**35.8 ± 55.4**	**0.029**	6.6 ± 9.6	13.3 ± 33.6*	0.170	0.141
**Onset-to-arrival time (n, %)**							
<1 hour	10 (36)	**15 (56)**	0.138	7 (30)	25 (30)*	0.753	0.383
<4 hours	20 (71)	**24 (89)**	0.100	14 (54)	51 (61)**	0.493	0.317
<12 hours	**23 (82)**	**24 (89)**	0.476	15 (58)*	56 (68)*	0.366	0.883
<24 hours	25 (89)	24 (89)	0.962	19 (73)	60 (72)	0.937	0.999
**Mortality**							
In-hospital death (n, %)	1 (4)	**5 (18)**	0.101	0 (0)	2 (2)**	1.000	0.661
30-day mortality (n, %)	1 (3)	**4 (15)**	0.193	0 (0)	1 (1)*	1.000	0.723

Data are presented as means ± SD or as medians with interquartile ranges (25th-75th percentiles). BP = blood pressure, CVD = cardiovascular disease, eGFR = estimated glomerular filtration rate, AST = aspartate aminotransferase, ALT = alanine aminotransferase, GPT = glutamic pyruvic transaminase, HDL = high-density lipoprotein, LDL = low-density lipoprotein. Items in bold indicate P < 0.05 between sexes in same type of dissection. * and ** indicate P < 0.05 and P < 0.01, respectively, between Stanford type A and B in same sex.

## Discussion

The current study demonstrated that elderly women had significantly a higher proportion of acute Stanford type A dissections, had larger maximal aortic dissection diameters, were less obese, had lower levels of TG, had higher levels of HDL-C, and a lower prevalence of metabolic syndrome compared to their male counterparts. These sex differences were not observed in patients with Stanford type B dissection, indicating that features of AAD may have changed in the recent aging Japanese society.

Although the IRAD reported that similar proportions of men and women underwent Stanford type A and B dissections^7)^, our results showed that women exhibited a higher prevalence of Stanford type A dissection compared to men, especially in the older populations. Mean ages of patients in the IRAD were 67 and 60 years old for women and men, respectively. A Chinese cohort study also reported similar sex proportions for Stanford type A and B dissection, with the study population having a mean age of 52 years old^3)^. However, both women and men in our study population was relatively older (71 and 66 years, respectively). Furthermore, a study reported that the proportion of women to men increased with age in patients receiving acute type A aortic dissection repair, with 65% of the patients aged 80 years or older being women^9)^. Taken together, these sex differences the proportion of those with Stanford type A and B dissection in our study may be attributed to the rapid change of the patient’s characteristics caused by the aging of society.

Women, who accounted for one thirds of the subjects with AAD, were older than men in our study population, a finding consistent with that presented in previous reports^1, 3)^. The IRAD reported showed that that female subjects were significantly older and more frequently presented with a history of hypertension^7)^. However, few reports have described the associations between AAD development and metabolic profiles, including BMI, precise lipid profiles, and diabetes profiles. In the current study, women with type A dissection were older, less obese, and had lower TG levels and higher HDL-C levels compared to men with type A dissection. Moreover, whole-cohort analysis showed that women had lower total protein and albumin levels compared to men. Recent studies, including our own, have reported that elderly patients with malnutrition have a poorer prognosis for cardiovascular diseases, such as heart failure^10, 11)^ and ischemic heart disease^12, 13)^. Furthermore, several reports have shown that malnourished patients with either type A or type B dissection experience worse outcomes^14, 15)^. These findings suggest that, in the current Japanese population, older and thinner women with malnutrition may represent a high-risk group for Stanford type A dissection.

The mechanism(s) for sex-related differences in AAD features still remain unclear. Male patients with bicuspid aortic valve and thoracic aortic aneurysm had been reported to exhibit more collagen degradation/disarray and smooth muscle cell loss compared to female patients^16)^, suggesting increased male susceptibility to AAD. Another study reported that aging female mice, but not male mice, showed activation of transforming growth factor (TGF)-β pathway, which is involved in the pathogenesis of aortic dissection and aneurysm^17-19)^, and reduced expression of C-type mannose receptor 1, a deficiency in which causes fibrosis due to reduced collagen uptake in left ventricle^20)^. Androgen and estrogen, sex hormones that have been known to decrease with age, are involved in the pathogenesis of fibrosis. Androgen exhibits anti-fibrotic effects by TGF-β pathway blockage^21)^, whereas estrogen inhibits angiotensin II-induced collagen synthesis^22, 23)^. These results suggest that TGF-β pathway activation and progression of fibrosis may contribute the occurrence of aortic dissection in elderly women. Consistent with this hypothesis, our data showed that the incidence of type A dissection increased in women aged 60 years and older. In contrast, among individuals aged 50 years or younger, the incidence was similar between women and men. In women with type B dissection, the maximum aortic diameter was positively associated with age, whereas no such association was observed in men. One possible explanation is the protective effect of female sex hormones in younger women; however, further investigation is needed.

Moreover, elderly women with type A dissection included herein showed poorer renal function. In addition, whole-cohort analysis showed lower total protein and albumin levels in women. Vitamin D deficiency, which is often observed in subjects with renal dysfunction and malnutrition, in elderly patients with AAD has been associated with increased levels of osteocalcin, which is involved in vascular calcification^24)^. Vitamin D deficiency also triggers rupture-prone abdominal aortic aneurysms through activation of osteopontin in the vascular wall of a mouse model^25)^. Although it remains unclear whether vascular calcification causes acute aortic disease^26, 27)^, vascular calcification induced by renal dysfunction and malnutrition may play a role in the development of thoracic AAD in elderly women. Further studies are warranted to clarify the relationship between the extent of vascular calcification and AAD development.

Previous reports have shown that women with AAD arrive late to the hospital^7)^. However, our study population showed no significant differences in onset-to-arrival time according to sex. Male patients with type A dissection had a significantly faster onset-to-arrival than those with type B dissection. Although the IRAD reported that only 27% of patients had been diagnosed within 4 hours from symptom onset^7)^, 34% and 66% of patients in this study arrived to our hospital within 1 and 4 hours from symptom onset and received emergency contrast-enhanced CT. The recent development of an effective and safe transfer system in Japan may have contributed to the disappearance of sex-related difference in onset-to-arrival time. In this study, patients who arrived 24 hours or more after symptom onset had significantly higher CRP levels than those who arrived within 24 hours. These findings suggest that earlier hospital presentation may contribute to improved clinical outcomes.

The IRAD demonstrated that women with AAD had higher mortality rates compared to men (women, 30.1%; men, 21.0%), especially among those with type A dissection and who had received surgical treatment (women, 31.9%; men, 21.9%)^7)^. Conversely, a study involving the Japanese population showed that male patients had significantly higher 30-day mortality (1.67 times compared to women) in patients with type A AAD^6)^. Other reports have also showed that women with type A aortic dissection repair had lower surgical mortality compared to men (men, 9.5%; women, 4.9%)^9)^. On the other hand, the IRAD showed no sex-related differences in mortality among patients with type B dissection (men, 12.3%; women, 14.4%)^7)^. Another study also found no sex-related differences in long-term clinical outcomes after thoracic endovascular aortic repair for Stanford type B dissection^28)^. The present study showed no significant difference in sex-related mortality among patients with both Stanford type A and B dissections. Compared to Western countries, relatively lower mortality rates from AAD had been reported from several Japanese cohort studies^4, 29, 30)^. Moreover, only a few subjects included herein died, with an in-hospital and 30-day mortality of only 4.9% and 3.7%, respectively. This difference in mortality rate compared to previous reports may have been attributed to differences in sex-related mortality.

This study has several limitations worth noting. Firstly, given the retrospective observational nature of this study, causal relationship between the sex-related clinical features and the development of AAD could not be determined. Secondly, this study had a relatively small sample size and was conducted at a single center. Although this study showed significant differences in the characteristic of AAD between women and men, larger prospective studies are needed to confirm our results.

Compared to men, elderly women exhibited a significantly higher prevalence of acute Stanford type A dissection and larger maximal aortic dissection diameters. Women with AAD also had lower BMI, total protein, and albumin levels than their male counterparts. Furthermore, among women, those with type A dissection had lower eGFR than those with type B dissection. These findings suggest that, in the context of Japan's aging society, older and leaner women with chronic kidney disease and malnutrition may constitute a high-risk population for Stanford type A dissection.

## Author contributions

HI, SK, TM, TM and HD analyzed and interpreted the patient data. HA, MK, MM, RS, SO, DO, YY, FO, KT, YS, MH, KY, MS, KS, TT and HI performed data collection. HI, SK and TM were a major contributor in writing the manuscript. All authors read and approved the final manuscript.

## Conflicts of interest statement

The authors declare that there are no conflicts of interest.
